# Accuracy of routinely collected hospital administrative discharge data and death certificate ICD-10 diagnostic coding in progressive supranuclear palsy and corticobasal syndrome: a systematic review and validation study

**DOI:** 10.1007/s00415-024-12280-w

**Published:** 2024-04-12

**Authors:** Diane M. A. Swallow, Carl E. Counsell

**Affiliations:** Institute of Applied Health Sciences, University of Aberdeen, Polwarth Building, Foresterhill, Aberdeen, AB25 2ZD UK

**Keywords:** Progressive supranuclear palsy, Corticobasal degeneration, Positive predictive value, Sensitivity, ICD-10 diagnostic codes, Death certificate

## Abstract

**Background:**

We conducted a systematic review to identify existing ICD-10 coding validation studies in progressive supranuclear palsy and corticobasal syndrome [PSP/CBS]) and, in a new study, evaluated the accuracy of ICD-10 diagnostic codes for PSP/CBS in Scottish hospital inpatient and death certificate data.

**Methods:**

Original studies that assessed the accuracy of specific ICD-10 diagnostic codes in PSP/CBS were sought. Separately, we estimated the positive predictive value (PPV) of specific codes for PSP/CBS in inpatient hospital data (SMR01, SMR04) compared to clinical diagnosis in four regions. Sensitivity was assessed in one region due to a concurrent prevalence study. For PSP, the consistency of the G23.1 code in inpatient and death certificate coding was evaluated across Scotland.

**Results:**

No previous ICD-10 validation studies were identified. 14,767 records (SMR01) and 1497 records (SMR04) were assigned the candidate ICD-10 diagnostic codes between February 2011 and July 2019. The best PPV was achieved with G23.1 (1.00, 95% CI 0.93–1.00) in PSP and G23.9 in CBS (0.20, 95% CI 0.04–0.62). The sensitivity of G23.1 for PSP was 0.52 (95% CI 0.33–0.70) and G31.8 for CBS was 0.17 (95% CI 0.05–0.45). Only 38.1% of deceased G23.1 hospital-coded cases also had this coding on their death certificate: the majority (49.0%) erroneously assigned the G12.2 code.

**Discussion:**

The high G23.1 PPV in inpatient data shows it is a useful tool for PSP case ascertainment, but death certificate coding is inaccurate. The PPV and sensitivity of existing ICD-10 codes for CBS are poor due to a lack of a specific code.

**Supplementary Information:**

The online version contains supplementary material available at 10.1007/s00415-024-12280-w.

## Introduction

Progressive supranuclear palsy (PSP) and corticobasal syndrome (CBS) are two related rare neurodegenerative diseases which can cause parkinsonism. Due to their rarity, single centre or regional studies are frequently limited by sample size. Routinely collected administrative health data sets contain large quantities of health information, often with near complete population coverage, spanning prolonged time periods. Linkage to such data sets therefore has the potential to greatly expand the scope of traditional cohorts, enabling cost-effective follow up, with reduced attrition, reporting or non-response bias. They may also provide a cost-effective means of case ascertainment for prevalence studies or to establish large disease specific national registers or data platforms, particularly for low prevalence conditions like PSP and CBS. In addition to clinical data collected during life, information from death certificates [1] is also used in epidemiological, public health and health services research to determine the incidence, prevalence and mortality for specific diseases in specified populations. Research using such data has been facilitated by the availability of a standard classification system for coding diagnoses, the International Classification of Diseases (ICD) [2], which is periodically updated, with version 10 (ICD-10) in use at the time of this study. Theoretically this should standardise data and facilitate the systematic recording, analysis, interpretation and comparison of morbidity and mortality data collected in different countries at different times.

Validation of coding accuracy is essential to evaluate the potential utility of routinely collected data sets in PSP/CBS, and to strengthen and appraise health care decisions based on any research conducted using ICD-10 diagnostic classifications. We therefore sought to: (i) systematically review the literature to identify and summarise existing studies evaluating the accuracy of ICD-10 codes in routinely collected data for PSP/CBS specifically and (ii) use the Scottish hospital inpatient data (Scottish Morbidity Record [SMR]) [3] and death certificate data from the National Health Service Central Register [NHSCR] [4] to: (a) determine the frequency and distribution of clinical diagnoses coded using candidate ICD-10 diagnostic codes; (b) determine the accuracy of ICD-10-coded diagnoses (PPV, sensitivity) in inpatient data compared to a reference clinical standard, and (c) determine the consistency of G23.1 discharge diagnostic coding for PSP in inpatient and death certificate data.

## Methods

### Systematic review

The rigorous search strategy used in a published systematic review of the diagnostic accuracy of routinely collected data in motor neuron disease [5] was modified and used to search MEDLINE (Ovid), EMBASE (Ovid), Web of Science (Thompson Reuters) and Cochrane (see Supplementary data). The reference lists of identified studies were also searched. To be eligible for inclusion, studies had to be full text (articles published only as abstracts or conference proceedings were excluded as methodological quality could not be determined) and report an original study that provided an analysis of the accuracy of specific ICD-10 diagnostic codes in PSP and CBS. Where available, the following information was extracted: first author, year of publication, routinely collected data source, reference or gold standard, and, measures of accuracy including sensitivity, PPV, specificity and negative predictive value (NPV).

### Evaluation of ICD-10 diagnostic codes

#### Data sets

Healthcare data for individual patients in Scotland are collected as a series of Scottish Morbidity Records (SMR). SMR01 is an episode-based data set describing all inpatients and day cases discharged from non-obstetric and non-psychiatric specialties. A record is generated when a patient completes an episode of inpatient or day case care, including discharge from hospital, transfer to another clinician, a change of specialty or death. SMR04 is similarly an episode-based record relating to all inpatients and day cases admitted to and discharged from Mental Health specialties. Diagnoses are coded in these data sets using ICD-10 diagnostic codes. The NHSCR holds a record for every patient registered with a Scottish GP, everyone born in Scotland since 1985 who has not been registered with a Scottish GP, and, patients formerly registered with a Scottish GP who died after 31 December 1992. The NHSCR data set is linked to death data collected in the General Register Office for Scotland Vital Events. Causes of death are also coded using ICD-10.

#### Diagnostic codes of interest

PSP has a specific ICD-10 diagnostic code, “G23.1 Progressive supranuclear ophthalmoplegia [Steele–Richardson–Olszewski]”. Previous research has shown that PSP patients have also been frequently erroneously assigned the “G12.22 Progressive Bulbar Palsy” code (G12.2 codes Motor Neuron Disease) [6]. There is no specific ICD-10 diagnostic code for corticobasal degeneration (CBD) or corticobasal syndrome (CBS), and no national guidance on non-specific coding use (personal communication). Potential non-specific coding options therefore additionally explored in this analysis are detailed in Table 1.

**Table 1:** Candidate ICD-10 diagnostic codes for PSP and CBS

ICD-10 code	Description
G23.1	Progressive supranuclear ophthalmoplegia [Steele-Richardson-Olszewski]
G12.22	Progressive Bulbar Palsy
G23.8	Other specified degenerative diseases of basal ganglia
G23.9	Degenerative disease of the basal ganglia, unspecified
G25.9	Extrapyramidal and movement disorder, unspecified
G31.0	Circumscribed brain atrophy (encompassing Pick’s disease and progressive isolated aphasia)
G31.8	Other specified degenerative diseases of the nervous system
F02.0	Dementia in Pick's disease

#### Prevalence data

SMR data were used as one method of case ascertainment in a national prevalence study which estimated the prevalence of PSP and CBS at regional and national levels in Scotland, UK. This study has been reported and described in detail elsewhere [7]. In brief, nationally, multiple methods of case ascertainment were used to identify cases, including clinician and nurse specialist referral, searches of ICD-10 diagnostic coding in SMR and patient self-referral. Two additional methods—searches of GP databases and unselected hospital correspondence—were restricted to NHS Grampian due to resource and time constraints. Identified cases were verified by clinical examination (where patients had also consented to participation in a concurrent prospective national cohort study, the Scottish PSP and CBS cohort [8]), medical record review, or through contact with an individual’s named consultant. Individuals were deemed prevalent cases if they had a diagnosis of PSP or CBS and were alive and resident within the study population on the prevalence day, December 31, 2018.

### Analysis

#### Frequency and distribution of clinical diagnoses coded using ICD-10 diagnostic codes G23.1, G25.9, G31.0, G31.8, G23.9, G23.8 and F02.0 in SMR01

As part of a preliminary evaluation of the utility of the SMR as a method of case ascertainment for the prevalence study detailed above, in NHS Grampian only, the clinical records of all individuals assigned the candidate ICD-10 diagnostic codes of interest, over a 2-year period from 1 April 2014 to 31 March 2016, were reviewed to evaluate the frequency and distribution of clinical diagnoses coded using each of these codes.

#### Accuracy of ICD-10-coded diagnoses (PPV, sensitivity) in SMR01 and SMR04 compared to a reference clinical standard in NHS Grampian, NHS Highland, NHS Orkney and NHS Shetland

All healthcare episodes from February 2011 to July 2019 in SMR01 and SMR04, with any of the ICD-10 codes of interest, in any diagnostic coding position (main diagnosis, secondary diagnoses), were extracted by the electronic Data Research and Innovation Service of the Information Services Division Scotland. Using postcodes and health board boundary data from the Scottish Government Ordnance Survey [9], the records of individual’s resident in NHS Grampian, NHS Highland, NHS Orkney and NHS Shetland (areas where we had access to the full medical record) were identified, and the relative frequency of healthcare episodes and individuals assigned each of the candidate ICD-10 diagnostic codes in these health boards compared to the total number of similar records from across NHS Scotland.

PPV and sensitivity were then calculated for each of the candidate ICD-10 diagnostic codes after we excluded individuals who died prior to 1 January 2018 (the start of prevalent period in the Scottish PSP and CBS prevalence study). The full electronic medical record (containing primary care referrals, hospital inpatient and outpatient correspondence and investigation results) of individuals with the candidate ICD-10 diagnostic codes in SMR01 and SMR04 were retrospectively reviewed. Reference standard clinical diagnoses were predominantly based on a clinician’s recorded diagnosis within these computerized medical records, further verified by clinical examination in people who had also consented to participation in a concurrent prospective national cohort study in PSP and CBS [8]. We estimated the PPV for two outcomes, a specific diagnosis of PSP or CBS, and, any parkinsonian disorder. The syndrome of parkinsonism was also evaluated as it is a key diagnostic milestone which, if identified, clinically narrows differential diagnoses, potentially minimising the impact of misdiagnosis or delayed diagnosis on the sensitivity of case finding of specific causes of the parkinsonian syndrome, including PSP/CBS [10]. The PPV was defined as the proportion of all coded cases that were confirmed from review of the medical record to be cases of PSP, CBS or parkinsonism, respectively. The small number of individuals in whom we were unable to access their medical record to assess their clinical diagnosis were excluded from analysis. Due to small numbers, confidence intervals for the PPV were estimated using the Wilson score interval. Sensitivity, defined as the proportion of all identified prevalent cases that were coded positive, was calculated in NHS Grampian only as this was the region where, due to active case ascertainment, we had most rigorously been able to minimise the risk of missing prevalent cases.

#### Consistency of G23.1 discharge diagnostic coding in SMR01 and SMR04 compared to NHSCR death certificate coding

In deceased individuals, coded with the G23.1 diagnostic code between February 2011 to July 2019 in SMR01 and SMR04, the consistency between discharge diagnostic coding and death certificate coding from linked vital events data was also evaluated.

The study was reviewed and approved by the Public Benefit and Privacy Panel for Health and Social Care (HSC-PBPP), NHS Scotland. All results were reported according to the modified Standards for Reporting of Diagnostic accuracy criteria [11].

## Results

### Systematic review

**Fig. 1:**
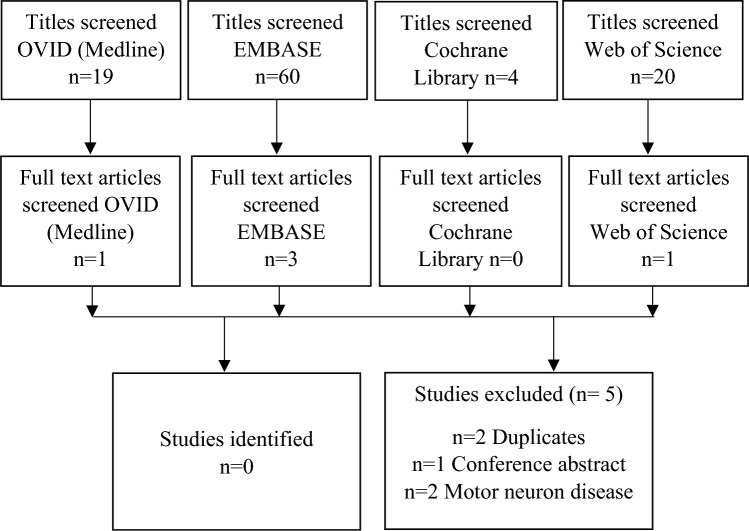
PRISMA flow diagram of search of accuracy of ICD-10 diagnostic coding in PSP and CBS

No studies were identified which evaluated the sensitivity or PPV of individual diagnostic ICD-10 codes for PSP or CBS (Fig. 1). Two of the full text reviewed articles [12, 13] highlighted the incorrect assignment of PSP patients to the “G12.22 Progressive Bulbar Palsy” code (G12.2 codes Motor Neuron Disease).

### Frequency and distribution of clinical diagnoses coded using ICD-10 diagnostic codes G23.1, G25.9, G31.0, G31.8, G23.9, G23.8 and F02.0 in SMR01

The clinical diagnoses in individuals assigned the candidate ICD-10 diagnostic codes are detailed in Table 2. All individuals with G23.1 code (*n* = 9) had a clinical diagnosis of PSP. Of 61 individuals with the G31.8 code, one individual had PSP listed amongst their differential diagnoses. CBS was not identified. The other clinical diagnoses assigned this code were a combination of parkinsonian (*n* = 4) and dementia (*n* = 49) syndrome diagnoses, as well as smaller number (*n* = 8) of other neurological diagnoses, detailed in Table 2. Few cases were assigned the remainder of the candidate ICD-10 codes. Two individuals, coded with both G31.0 and F02.0, had a predominantly frontotemporal dementia syndrome with potentially suggestive clinical features (postural instability, symmetrical rigidity, frontal behavioural–spatial syndrome) of PSP or CBS, though neither had been given these diagnoses. No cases assigned the G25.9, G23.9, or G23.8 ICD-10 diagnostic codes had a clinical diagnosis of PSP or CBS.

**Table 2:** Frequency and distribution of clinical diagnoses assigned ICD-10 codes G231, G259, G310, G318, G239, G238 and F020 in SMR01 in NHS Grampian

ICD-10 code	Episodes	Individual cases	Diagnoses		Individuals potentially PSP/CBS
G23.1	19	9	**PSP**	**9**	9 (100%)
G25.9	1	1	Functional movement disorder	1	0 (0%)
G31.0	7	4	Hereditary leukodystrophy	1	2 (50%)
			**FTD (plus postural instability, symmetrical rigidity)**	**1**	
			**FTD (but possible fronto-behaviour spatial syndrome)**	**1**	
			Global atrophy due alcohol misuse	1	
G31.8	108	61	Parkinsonian diagnoses		8 (13.1%)
			PD (no significant dementia)	2	
			**Parkinsonian syndrome**	**1**	
			**?PSP/MND**	**1**	
			Cognitive impairment/dementia		
			MCI secondary cerebrovascular disease	1	
			Vascular dementia	2	
			DLB	27	
			**Labelled DLB but atypical**	**5**	
			PDD	7	
			PDD or DLB	2	
			Mixed DLB/vascular dementia	1	
			Mixed AD/vascular dementia	1	
			Mixed dementia	1	
			DLB or vascular dementia	1	
			Aetiology dementia unclear	1	
			Other diagnoses		
			Degenerative disease /spondylitis	1	
			RRMS, occipital meningioma	1	
			Spinocerebellar degeneration	3	
			MND	1	
			**Diagnosis unclear (ataxic, dysarthric)**	**1**	
			Misdiagnosis DLB	1	
G23.9	0	0			
G23.8	1	1	SAH/SDH	1	0 (0%)
F02.0	6	3	Hereditary leukodystrophy	1	2 (66.6%)
			**FTD (plus postural instability, symmetrical rigidity)**	**1**	
			**FTD (but possible fronto-behaviour spatial syndrome)**	**1**	
Total	136	76			20
Adjusted total	130^a^	73^b^			16^c^ (23.7%)

Items in bold are possible PSP/CBS cases

*AD *Alzheimer’s disease, *DLB* dementia with Lewy bodies, *FTD* fronto-temporal dementia, *MND* motor neuron disease, *PD * Parkinson’s disease, *PDD* Parkinson’s disease dementia, *PSP* progressive supranuclear palsy, *RRMS* relapsing remitting multiple sclerosis, *SAH* subarachnoid haemorrhage, *SDH* subdural haemorrhage

^a^Six with two ICD-10 codes, ^b^three with two ICD-10 codes, ^c^two with two ICD-10 codes

### Accuracy of ICD-10-coded diagnoses (PPV, sensitivity) in SMR01 and SMR04 compared to a reference clinical standard in NHS Grampian, NHS Highland, NHS Orkney and NHS Shetland

14,767 records (including *n* = 2119 in selected health boards) in SMR01, and 1497 records (including 1901 in selected health boards) in SMR04 across NHS Scotland were assigned the candidate ICD-10 diagnostic codes between February 2011 and July 2019 (Table 3). The most frequently assigned ICD-10 codes in SMR01 were “G12.2-motor neuron disease”, and “G31.8-other specified degenerative diseases of the nervous system”. Apart from codes G31.0, G31.8 and F02.0, very few discharges in SMR04 were coded with the remainder of the tested ICD-10 codes. Only five discharges across Scotland, for example, in SMR04 were assigned the G23.1 code.

**Table 3:** Total number of records and individuals coded with ICD-10 codes G23.1, G12.2, G23.8, G23.9, G25.9, G31.0 and G31.8 from February 2011 to July 2019 in selected health boards (NHS Grampian, Highland, Orkney and Shetland) and across Scotland in SMR01 and SMR04

	SMR		Number of episodes and individual cases with ICD-10 code								Total population^†^
			G23.1	G12.2	G23.8	G23.9	G25.9	G31.0	G31.8	F02.0	
Selected regional health boards	SMR01	Records	158	1192	25	7	80	14	643	0	951,530
		Individual cases	53	357	17	5	34	12	295	0	
	SMR04	Records	0	3	1	0	2	50	90	44	
		Individual cases	0	2	1	0	1	38	74	34	
NHS Scotland	SMR01	Records	922	7855	276	35	900	231	4548	0	5,438,100
		Individual cases	279	2169	137	19	333	108	1740	0	
	SMR04	Records	5	11	4	0	10	426	673	368	
		Individual cases	5	10	4	0	9	294	441	252	

^†^Mid-year population estimates in Scotland on 30 June 2018 (accessed from the National Records of Scotland)

The PPVs of each of the candidate ICD-10 codes for PSP, CBS and parkinsonism (a key diagnostic milestone which narrows differential diagnoses) in SMR01 and SMR04 are shown in Table 4. Within selected health boards in SMR01, overall, the PPV of the G23.1 diagnostic code for both parkinsonism and PSP was extremely high at 1.00 (95% CI 0.93, 1.00) (PSP cases assumed to be parkinsonian), with all cases assigned the code having a corresponding clinical diagnosis of PSP. Despite the availability of a specific code for PSP, 21 cases were identified amongst those assigned the G12.2 diagnostic code (PPV of 0.06 [95% CI 0.04, 0.09]). Of the remaining ICD-10 codes considered, one case with a clinical diagnosis of PSP was identified for each of the G31.8 and G23.9 codes. For CBS, only the G23.9 and G31.8 diagnostic codes identified individuals with a corresponding clinical diagnosis. Unsurprisingly given the lack of a specific diagnostic code for CBS, the PPV of these diagnostic codes was much lower than that achieved by the G23.1 code for PSP, with PPVs of 0.20 (95% CI 0.04, 0.62) and 0.02 (95% CI 0.01, 0.05), respectively. Aside from the high PPV of the G23.1 code for parkinsonism (in that all cases of PSP were assumed to be parkinsonian), the PPV of the G31.8 code for parkinsonism was 0.55 (95% CI 0.49, 0.61), the majority of true positive cases having a diagnosis of dementia with Lewy bodies (DLB) or PD dementia (PDD). A small number of cases with parkinsonism were assigned the G23.8, G23.9 and G25.9 codes.

**Table 4:** Positive predictive values for candidate ICD-10 codes for PSP, CBS and parkinsonism in SMR01 in NHS Grampian, NHS Highland, NHS Orkney and NHS Shetland

ICD-10 code	Number of individuals with ICD-10 code	PSP			CBS			Parkinsonism		
		TP	FP	PPV (95% CI)	TP	FP	PPV (95% CI)	TP	FP	PPV (95% CI)
SMR01										
G23.1	50^a^	50	0	1.00 (0.93, 1.00)	0	50	0.00 (0.00, 0.07)	50	0	1.00 (0.93, 1.00)
G12.2	334^b^	21	313	0.06 (0.04, 0.09)	0	334	0.00 (0.00, 0.01)	21	313	0.06 (0.04, 0.09)
G23.8	16^c^	0	16	0.00 (0.00, 0.19)	0	16	0.00 (0.00, 0.19)	2	14	0.13 (0.03, 0.36)
G23.9	5^†^	1^†^	4	0.20 (0.04, 0.62)	1^†^	4	0.20 (0.04, 0.62)	2	3	0.40 (0.12, 0.77)
G25.9	31^d^	0	31	0.06 (0.00, 0.11)	0	31	0.06 (0.00, 0.11)	3	28	0.14 (0.03, 0.25)
G31.0	12	0	12	0.00 (0.00, 0.24)	0	12	0.00 (0.00, 0.24)	0	12	0.00 (0.00, 0.24)
G31.8	271^e^	1	270	0.00 (0.00, 0.02)	6	265	0.02 (0.01, 0.05)	150	121	0.55 (0.49, 0.61)
SMR04										
G23.1,12.2, 23.8 & 25.9	4	1	3	0.37 (0.05, 0.70)	0	4	0.24 (0.00, 0.49)	1	3	0.37 (0.05, 0.70)
G31.0	35^f^	1*	34	0.08 (0.01, 0.15)	0	35	0.05 (0.00, 0.10)	3	32	0.13 (0.03, 0.22)
G31.8	72^ g^	0	72	0.00 (0.00, 0.05)	0	72	0.00 (0.00, 0.05)	66	6	0.92 (0.83, 0.96)
F02.0	33^ h^	1*	32	0.03 (0.01, 0.15)	0	33	0.00 (0.00, 0.10)	2^ǂ^	31	0.06 (0.02, 0.20)

*CBS* corticobasal syndrome, *CI* confidence interval, *FP* false positive, *PPV* positive predictive value, *PSP* progressive supranuclear palsy, *SMR* Scottish Morbidity Record, *TP* true positive

^a^Excluding 3 individuals unable to access record, ^b^excluding 23 individuals unable to access record, ^c^excluding 1 unable to access record, ^d^excluding 3 unable to access record, ^e^excluding 24 individuals unable to access record, ^f^excluding 7 individuals unable to access record, ^g^excluding 3 individuals unable to access record, ^h^excluding 4 individuals unable to access record. ^†^1 individual clinically indistinguishable PSP/CBS, *n = 1 with no formal diagnosis but frontal cognitive difficulties, bulbar features, falls (RIP prior to diagnosis) ^₼^*n* = 1 with no formal diagnosis but frontal cognitive difficulties, bulbar features, falls (RIP prior to diagnosis), DLB *n* = 1, FTD with parkinsonism *n* = 1. ^**ǂ**^*n* = 1 no formal diagnosis but frontal cognitive difficulties, bulbar features, falls (RIP prior to diagnosis), FTD with parkinsonism *n* = 1. Confidence interval Wilson score method http://www.statskingdom.com/41_proportion_confidence_interval.ht

Within SMR04, no individuals from the four health boards of interest were assigned the G23.1 diagnostic code for PSP. Indeed, across all tested ICD-10 codes within SMR04, only three individuals with PSP were identified. No cases with a clinical diagnosis of CBS were identified across all of the ICD-10 discharge diagnostic codes. The G31.8 code had a high PPV for parkinsonism, predominantly due to cases of DLB/PDD (*n* = 66/72 cases), the remainder of coded cases having a clinical diagnosis of vascular dementia (*n* = 2), fronto-temporal dementia (FTD) (*n* = 2) or unspecified dementia (*n* = 2). The majority of cases assigned the G31.0 and F02.0 diagnostic codes had FTD (*n* = 32/35 coded cases for G31.0 and *n* = 30/33 for F02.0).

Using the regional prevalence figures [7], the sensitivity of each of the diagnostic codes for detecting PSP or CBS is shown in Table 5. 25 PSP and 12 CBS cases were identified in NHS Grampian on the prevalence day. Of CBS cases, 2 cases were coded within SMR01 (both G31.8 code). No cases were identified in SMR04. Of 25 PSP cases, 13 were coded in SMR01 (all G23.1 with one case additionally coded with G12.2). No cases were identified in SMR04. The sensitivity of the G23.1 diagnostic code for detecting prevalent PSP cases was therefore 0.52 (95% CI 0.33, 0.70) while the sensitivity of the G12.2 code was 0.04 (95% CI 0.01, 0.20). Only the G31.8 diagnostic code identified CBS cases but with a low sensitivity (0.17, 95% CI 0.05, 0.45).

**Table 5:** Sensitivity of ICD-10 diagnostic coding in SMR01 for PSP or CBS

ICD-10 code	PSP *n* = 25			CBS *n* = 12		
	TP	FN	Sensitivity	TP	FN	Sensitivity
G23.1	13	12	0.52 (0.33, 0.70)	0	12	0.00 (0.00, 0.24)
G12.2	1	24	0.04 (0.01, 0.20)	0	12	0.00 (0.00, 0.24)
G23.8	0	25	0.00 (0.00, 0.13)	0	12	0.00 (0.00, 0.24)
G23.9	0	25	0.00 (0.00, 0.13)	0	12	0.00 (0.00, 0.24)
G25.9	0	25	0.00 (0.00, 0.13)	0	12	0.00 (0.00, 0.24)
G31.0	0	25	0.00 (0.00, 0.13)	0	12	0.00 (0.00, 0.24)
G31.8	0	25	0.00 (0.00, 0.13)	2	10	0.17 (0.05, 0.45)

*PSP* progressive supranuclear palsy, *CBS* corticobasal syndrome, *TP* true positive, *FN* false negative

### Consistency of G23.1 discharge diagnostic coding in SMR01 and SMR04 compared to NHSCR death certificate coding

224 of 284 individuals identified across Scotland from February 2011 to July 2019 with an SMR01 or SMR04 code of G23.1 for PSP had died by July 2019. Of these 77 (34.4%) individuals also had G23.1 on their death certificate. 101 (45.1%) were assigned the G12.2 code, 24 (10.7%) none of the tested ICD-10 codes, and three (1.3%) the G31.8 code. In one (0.4%) individual their death certificate data was coded with both G12.2 and G31.8.

## Discussion

Validation of administrative data coding has been identified as a priority in health services research [14]. While coding accuracy has been reported for other neurological disorders [5, 15], including PD and parkinsonism more broadly [16], to our knowledge this is the first study to explore the accuracy of individual ICD-10 diagnostic codes for PSP and CBS specifically.

Compared to the SMR01 data set, and despite the burden of cognitive and behavioural features in PSP/CBS, the SMR04 data set has limited value, with low absolute numbers of PSP and CBS cases. We have demonstrated wide variation in the performance of the tested ICD-10 codes for PSP/CBS, reflecting the heterogeneity of the individual codes assessed. For PSP, while the PPV for G23.1 was 100%, its sensitivity was rather low with just over half (52%) of prevalent PSP cases identified. The sensitivity of the SMR01 data set in general, however, does appear superior to death certificate data in that only 34.4% of deceased hospital-coded G23.1 cases also had this coding on their death certificate. Whilst this difference may have been appropriate if their diagnosis had been revised during life or if PSP had not contributed to their death, it seems unlikely this explains all the difference. Moreover, many (45.1%) were erroneously assigned an incorrect code: G12.2 for progressive bulbar palsy (a variant of motor neuron disease) as opposed to progressive supranuclear palsy. Indeed, we have found subsequently that all individuals recruited to an incident population based cohort of parkinsonism in the North–East of Scotland (the PINE study) [17] who had a textual diagnosis of PSP on their death certificate (*n* = 31) were wrongly assigned the G12.2 diagnostic code (unpublished). For CBS, all tested ICD-10 codes had poor PPVs, unsurprising given the lack of a specific ICD-10 code for CBS. While the G31.8 code, specified as the preferred code for DLB, was most frequently assigned, both its PPV and sensitivity for CBS was low (2% and 17%), even when considering the upper boundary of calculated 95% confidence intervals. Where maximising sensitivity is a priority, cases with FTD assigned the G31.0 or F02.0 ICD-10 codes may have motor features in keeping with subtypes of CBS (or PSP).

From our systematic review we identified no studies which previously assessed the accuracy of ICD-10 coding on routinely collected data specifically for PSP or CBS. One previous study from 2005, using ICD-9 coding found a sensitivity of 31% for the specific PSP code on death certificates [18]. A small number of existing studies have evaluated the accuracy of textual diagnoses in death certification in PSP, with the diagnosis reported on between 40.0 and 65% [17–20] death certificates, with no variation according to sex, age, or place of death in one study [18].

Implications for practice depend on the intended use of coded data. If no further assessment is envisaged, the high PPV for the G23.1 code indicates most cases identified using the G23.1 code would truly have PSP, minimising bias in effect estimates due to misclassification of cases. However due to the lower demonstrated sensitivity of the same code, cohorts established in such a manner may be underpowered (due to the rarity of PSP) and may also introduce bias if patients who are not coded systematically differ from coded cases. Low sensitivity also has important implications for studies where inclusiveness is a priority, such as prevalence studies. The omission of PSP or CBS from death certificates may lead to an underestimation of disease-specific mortality, as well as limit an assessment of co-morbidities and indirect deaths in the PSP and CBS population.

Several mechanisms may result in inaccuracies in routinely collected data. Due to the absence of a biomarker with adequate sensitivities and specificities, misclassification of the reference standard (clinical diagnosis) is possible. Where reference standards are dependent on medical record review, incomplete documentation may result in coding errors. Administrative miscoding, coding errors, differences in coding practice or selection coding rules, such as the identified erroneous application of the G12.2 code for PSP [6], may also lead to errors. Accuracy is also clearly dependent on the availability of a disease specific code, a key limitation in CBS. The omission of PSP from death certificates may also arise by diagnostic and administrative mechanisms (such as selection and modification rules) and may also be omitted if the cause of death was completely unrelated to PSP, so that it was neither a direct cause nor a contributing factor to death.

Our study has several strengths. Due to a concurrent high-quality prevalence study, with rigorous, overlapping methods of case ascertainment, we were able to evaluate the sensitivity of various ICD-10 codes within hospital inpatient data as well as PPV estimates as measures of accuracy which is infrequently determined in coding accuracy validation studies. With increasing research governance limits on the use of non-specific codes due to perceived threats to patient confidentiality, this greater understanding of the PPV of individual codes will facilitate a precise selection of ICD-10 diagnostic codes and provide evidence to justify the selected use of such patient identifiable information. Other strengths include the inclusion of both non-obstetric and non-psychiatric specialties (SMR01) and psychiatric (SMR04) inpatient data sets to examine coding accuracy and well as access to the medical record to determine clinical diagnoses. The study also has several limitations. Misdiagnosis and delayed diagnosis in PSP and CBS may result in misclassification of the reference standard (clinical diagnosis). While we assessed the role of individual codes in detecting prevalent cases, it would be useful to compare diagnostic codes and clinical diagnosis at the time of each individual healthcare episode. While a proportion of cases with PSP/CBS were examined (*n* = 11 in selected NHS health boards) due to a concurrent cohort study, reference clinical diagnoses were largely based on medical record review rather than an independent clinical evaluation. While we calculated the sensitivity of SMR admission discharge data to identify PSP/CBS using prevalence data as the ‘true’ number of people with PSP and CBS in the population, the true sensitivity may be lower as cases in the population may be undiagnosed/misdiagnosed as something else. Finally, not all PSP/CBS cases will be admitted to hospital, particularly if they are early in their illness, a limitation of the inpatient SMR data sets.

Advancement in this area relies on improving clinical diagnosis and biomarker development, greater precision in medical record documentation [21], greater specificity of the ICD codes or agreement on non-specific code use, and coder training. A welcome development is that the ICD version 11 has a reformulated chapter structure and indexing system including a specific code for CBS (8A00.1Y) [22]. Implementation and familiarisation with the new indexing system will, however, take time. Previous studies have suggested that combining multiple data sets [16], or creating algorithms using a variety of variables within data sets [23] rather than solely relying on diagnostic coding may improve diagnostic accuracy. The outpatient data set of the SMR (SMR00) is still in the process of development but should further improve sensitivity for PSP/CBS. An evaluation of primary care READ codes [24] may be an interim useful source for identifying PSP/CBS cases. Prescribing data as a proxy for PSP/CBS cases will be more limited as not all PSP/CBS cases will have trialled anti-parkinsonian medications and numbers will be dwarfed by PD cases given its higher disease prevalence.

In conclusion, we have assessed the accuracy of ICD-10 codes for identifying PSP and CBS in both general and psychiatric inpatient data sets. At present, the high PPV for G23.1 code in SMR01 suggests that it is a useful tool for PSP case ascertainment for prospective population-based registers or epidemiologic studies, provided that active surveillance and multiple sources of case ascertainment are used to maximise case ascertainment. Unfortunately, both the PPV and sensitivity of a range of candidate ICD-10 codes for CBS are poor. Several highlighted areas of future development and research hold potential for improving the diagnostic accuracy and utility of ICD-coded data sets.

### Supplementary Information

Below is the link to the electronic supplementary material.

Supplementary file1 (DOCX 17 KB)

## Data Availability

Anonymised data relevant to the analyses within this article can be shared at the request of qualified investigators to replicate presented analyses.
