# Supplementary material for: Accuracy of routinely collected hospital administrative discharge data and death certificate ICD-10 diagnostic coding in progressive supranuclear palsy and corticobasal syndrome: a systematic review and validation study
Source: J Neurol. 2024 Apr 12;271(6):2929–37. doi: 10.1007/s00415-024-12280-w (PMC11136796; doi:10.1007/s00415-024-12280-w)
Supplement: Supplementary file 1 — Supplementary file1 (DOCX 17 KB) [file 415_2024_12280_MOESM1_ESM.docx]

**Supplementary data**

**Systematic review search strategy**

**1. OVID Medline 23/4/19**

1."International Classification of Diseases"/ or "international classification of diseas*".mp. or "ICD ten*".mp. or ICD10.mp. or "ICD 10".mp. or "ICD 9".mp. or ICD9.mp. or "ICD nine".mp. or ICD-9-CM.mp. or ICD-10-CM.mp. or "administrat* data".mp. or "medical record*".mp. or "health information*".mp. or claim*.mp. or "hospital discharge*".mp. or "inpatient discharge*".mp. or "hospital episode*".mp. or "hospital episode statistics".mp. or "scottish morbidity record*".mp. or SMR*.mp. or "patient episode database for wales".mp. or PEDW.mp. or coding.mp. or code*.mp. or exp Clinical Coding/ or "medical record review".mp. or exp Information Systems/ or exp Medical Records/ or exp medical records systems, computerized/ or exp electronic health records/ or exp Electronic Health Records/exp or Primary Health Care/exp or general practice/ or exp family practice/ or "read cod*".mp. or exp Patient Discharge/ or exp Patient Discharge Summaries/ or exp Hospital Records/ or exp Health Services Research/ or "physician claims".mp. or "death certificate*".mp. or exp death certificates/ or exp hospital records/ or "death registration*".mp. or case ascertainment.mp. or medicare or exp health insurance/ or exp Outpatients/ or "outpatient data".mp.

2.(sensitivity or specificity).mp. or exp "sensitivity and specificity"/ or ((pre-test or pretest) adj probability).mp. or exp "Predictive Value of Tests"/ or "predictive value*".mp. or "likelihood ratio*".mp. or exp validation studies/ or "validation stud*".mp. or "positive predictive value".mp. or exp "reproducibility of results"/ or "reproducibility of results".mp. or "positive predictive value".mp. or "negative predictive value".mp. or validity.mp. or reproducibility.mp. or accuracy.mp. or agreement.mp. or validation.mp. or algorithm*.mp. or exp algorithms/ or (identif* adj3 dement*).ti,ab. or (detect* adj3 dement*).ti,ab. or (ROC or "receiver operat*").ab. or sROC.ab. or Area Under Curve/

3. exp Supranuclear Palsy, Progressive/ or progressive supranuclear pals$.tw,mp. or Richardson$ syndrome.tw,mp. or Richardson's Syndrome/ or Richardsons syndrome/ or steele richardson olszewski disease.tw,mp. or steele richardson olszewski disease/ or steele richardson olszewski syndrome.tw,mp. or steele richardson olszewski syndrome/ or steele-richardson-olszewski disease.tw,mp. or steele-richardson-olszewski disease/ or steele-richardson-olszewski syndrome.tw,mp. or steele-richardson-olszewski syndrome/ or exp steele richardson olszewski syndrome/ or PSP-P.tw. or PSP-CBS.tw. or PSP-PAGF.tw. or PSP-RS.tw. or (progressive supranuclear palsy adj2 parkinsonism).tw,mp. or Pure akinesia with gait freezing.tw,mp. or PSP-F.tw. or PSP-SL.tw. or PSP-PI.tw. or PSP-OM.tw. or PSP-PGF.tw. or Corticobasal Degeneration.tw,mp or Corticobasal Degeneration/ or Corticobasal Syndrome.tw,mp or Corticobasal Syndrome/

4. 1 and 2 and 3

**2. EMBASE 23/4/19**

1. "International Classification of Diseases"/ or "international classification of diseas*".mp. or "ICD ten*".mp. or ICD10.mp. or "ICD 10".mp. or "ICD 9".mp. or ICD9.mp. or "ICD nine".mp. or ICD-9-CM.mp. or ICD-10-CM.mp. or "administrat* data".mp. or "medical record*".mp. or "health information*".mp. or claim*.mp. or "hospital discharge*".mp. or "inpatient discharge*".mp. or "hospital episode*".mp. or "hospital episode statistics".mp. or "scottish morbidity record*".mp. or SMR*.mp. or "patient episode database for wales".mp. or PEDW.mp. or coding.mp. or code*.mp. or exp Clinical Coding/ or "medical record review".mp. or exp Information Systems/ or exp Medical Records/ or exp medical records systems, computerized/ or exp electronic health records/ or exp Electronic Health Records/exp or Primary Health Care/exp or general practice/ or exp family practice/ or "read cod*".mp. or exp Patient Discharge/ or exp Patient Discharge Summaries/ or exp Hospital Records/ or exp Health Services Research/ or "physician claims".mp. or "death certificate*".mp. or exp death certificates/ or exp hospital records/ or "death registration*".mp. or case ascertainment.mp. or medicare.mp. or exp health insurance/ or exp Outpatients/ or "outpatient data".mp. [mp=title, abstract, heading word, drug trade name, original title, device manufacturer, drug manufacturer, device trade name, keyword, floating subheading word, candidate term word]

2. (sensitivity or specificity).mp. or exp "sensitivity and specificity"/ or ((pre-test or pretest) adj probability).mp. or exp "Predictive Value of Tests"/ or "predictive value*".mp. or "likelihood ratio*".mp. or exp validation studies/ or "validation stud*".mp. or "positive predictive value".mp. or exp "reproducibility of results"/ or "reproducibility of results".mp. or "positive predictive value".mp. or "negative predictive value".mp. or validity.mp. or reproducibility.mp. or accuracy.mp. or agreement.mp. or validation.mp. or algorithm*.mp. or exp algorithms/ or (identif* adj3 dement*).ti,ab. or (detect* adj3 dement*).ti,ab. or (ROC or "receiver operat*").ab. or sROC.ab. or Area Under Curve/

3. exp Supranuclear Palsy, Progressive/ or progressive supranuclear pals$.tw,mp. or Richardson$ syndrome.tw,mp. or Richardson's Syndrome/ or Richardsons syndrome/ or steele richardson olszewski disease.tw,mp. or steele richardson olszewski disease/ or steele richardson olszewski syndrome.tw,mp. or steele richardson olszewski syndrome/ or steele-richardson-olszewski disease.tw,mp. or steele-richardson-olszewski disease/ or steele-richardson-olszewski syndrome.tw,mp. or steele-richardson-olszewski syndrome/ or exp steele richardson olszewski syndrome/ or PSP-P.tw. or PSP-CBS.tw. or PSP-PAGF.tw. or (progressive supranuclear palsy adj2 parkinsonism).tw,mp. or Pure akinesia with gait freezing.tw,mp. or PSP-F.tw. or PSP-SL.tw. or PSP-PI.tw. or PSP-OM.tw. or PSP-PGF.tw.

4. 1 and 2 and 3

**3. Cochrane 23/4/19**

1. “international classification of diseases” OR “ICD*” OR “administrat* data” OR “medical record*” OR “health information” OR claim* OR “hospital discharge*” OR “inpatient discharge*” OR “hospital episode*” OR “hospital episode statistics” OR “scottish morbidity record” OR “patient episode database for wales” OR coding OR code* OR “medical record*” OR “electronic health record*” OR “hospital record*” OR medicare OR “health insurance”

2. “positive predictive value” OR “negative predictive value” OR accuracy OR sensitivity OR specificity OR validity

3. "progressive supranuclear palsy" OR "corticobasal ganglionic degeneration"

4. 1 AND 2 AND 3

**4. Web of Science 23/4/19**

1. TS=(“international classification of diseases” OR “ICD*” OR “administrat* data” OR “medical record*” OR “health information” OR claim* OR “hospital discharge*” OR “inpatient discharge*” OR “hospital episode*” OR “hospital episode statistics” OR “scottish morbidity record” OR “patient episode database for wales” OR coding OR code* OR “medical record*” OR “electronic health record*” OR “hospital record*” OR medicare OR “health insurance”)

2. TS=(“positive predictive value” OR “negative predictive value” OR accuracy OR sensitivity OR specificity OR validity)

3. TS=("progressive supranuclear palsy" OR “corticobasal degeneration”)

4. #3 AND #2 AND #1
